# The Inclusion of Supercritical Fluid Extract of *Ulva lactuca* in the Diet of Common Carp (*Cyprinus carpio*) Enhances Physiological Resilience and Antioxidant Status During Therapeutic-Dose Antibiotic Administration

**DOI:** 10.3390/antiox15091171

**Published:** 2026-09-15

**Authors:** Alina Nicoleta Macoveiu (Dobre), Liliana Mihalcea, Dana Moraru, Nicoleta Bălan, Mirela Crețu, Angelica Docan, Iulia Grecu, Cristian Rîmniceanu, Daniela Ionela Istrati, Floricel Maricel Dima, Lorena Dediu

**Affiliations:** 1 Faculty of Food Science and Engineering, Dunarea de Jos University of Galati, 111 Domneasca Street, 800201 Galați, Romania; adobre14@gmail.com (A.N.M.); dana.moraru@ugal.ro (D.M.); nicoleta.balan@ugal.ro (N.B.); angelica.docan@ugal.ro (A.D.); iulia.grecu@ugal.ro (I.G.); cristian.rimniceanu@ugal.ro (C.R.); daniela.istrati@ugal.ro (D.I.I.); 2 Institute of Research and Development for Aquatic Ecology, Fishing and Aquaculture, 54 Portului Street, 800211 Galați, Romania; dima.floricel.maricel@asas-icdeapa.ro; 3 Transborder Faculty, Dunărea de Jos University of Galați, 47 Domnească Street, 800008 Galați, Romania; mirela.cretu@ugal.ro; 4 Faculty of Engineering and Agronomy in Braila, Dunarea de Jos University of Galati, 29 Calea Calarașilor Street, 810017 Brăila, Romania

**Keywords:** florfenicol, oxytetracycline, macroalgae, functional feed, aquaculture

## Abstract

The widespread use of antibiotics in aquaculture has raised concerns regarding adverse physiological effects on fish, creating a need for dietary strategies that enhance fish resilience during antibiotic administration and support animal welfare under therapeutic treatment conditions. This study evaluated, for the first time, a phytochemically characterized supercritical fluid extract of *Ulva lactuca* (Ul-SFE), rich in flavan-3-ols (epigallocatechin, epicatechin gallate) and other phenolics, as a dietary supplement in common carp (Cyprinus carpio). Juvenile carp (n = 360; 20.20 ± 0.13 g) received a control or Ul-SFE-supplemented diet (50 mg kg^−1^) for 10 weeks, followed by 10 days of florfenicol or oxytetracycline exposure at therapeutic doses. Growth, plasma biochemistry, oxidative stress, and innate immunity were assessed. Ul-SFE significantly improved FCR, SGR, and PER during growth. Under antibiotic challenge, both florfenicol and oxytetracycline affected growth performance and biochemical status, though with different predominant patterns: florfenicol exposure was mainly characterized by reduced growth performance, while oxytetracycline exposure was mainly characterized by biochemical disturbance—reduced total protein and globulin, elevated glucose, ALT, and lipid peroxidation, and impaired antioxidant/immune status. Ul-SFE consistently attenuated these effects, reducing malondialdehyde by 24–30% and increasing antioxidant capacity by 5–7%; its protective effect on lysozyme was strongly diet-dependent (Diet × Antibiotic, *p* < 0.001), ranging from a 20% increase without antibiotics to negligible under oxytetracycline. These findings show Ul-SFE improves feed utilization and confers partial, treatment-dependent resilience against antibiotic-induced stress, supporting its potential as a functional feed additive in aquaculture.

## 1. Introduction

Aquaculture is undergoing rapid intensification to meet increasing global demand, exposing farmed fish to chronic physiological stress from overcrowding and water quality deterioration, which suppresses immune function and increases susceptibility to bacterial disease [1,2,3,4], driving the widespread use of therapeutic and prophylactic antibiotics [5,6].

Beyond direct therapeutic administration, fish reared in open aquaculture systems and net cages are also chronically exposed to antibiotic residues present in surface waters as environmental contaminants, originating from agricultural runoff, wastewater discharge, and upstream aquaculture and livestock operations [7,8]. Unlike controlled therapeutic dosing, this passive exposure occurs continuously and often as complex antibiotic mixtures, compounding the physiological burden already imposed by intensive farming conditions and providing an additional, environmentally relevant rationale for investigating strategies that support fish resilience to antibiotic-associated stress.

Florfenicol (FF) and oxytetracycline (OTC) are among the most widely used antibiotics in aquaculture, owing to their broad-spectrum activity; FF inhibits bacterial protein synthesis via ribosomal interference [9,10], while OTC blocks aminoacyl-tRNA binding to the 30S ribosomal subunit [11,12]. Despite their therapeutic efficacy, antibiotic administration in fish has been associated with compromised growth performance [13], disrupted intestinal microbial homeostasis [14,15], suppressed innate immune responses [16], altered mitochondrial function [17], and oxidative stress [18,19], potentially resulting in metabolic dysfunction and cellular damage [20].

Consequently, scientific attention has turned toward complementary nutritional strategies capable of mitigating these adverse effects while preserving therapeutic efficacy [21], with functional ingredients such as phytobiotics, probiotics, and algae-derived compounds shown to alleviate antibiotic-induced physiological disturbances and support disease resilience [22,23,24]. Marine macroalgae have attracted attention due to their richness in polyphenols, sulfated polysaccharides, pigments [25,26], and other bioactive compounds with antioxidants, immunomodulatory and hepatoprotective properties [27,28]. Among marine macroalgae, species of the genus Ulva have emerged as promising sources of functional feed ingredients. Both Ulva biomass and its conventional solvent extracts have been reported to improve growth performance, enhance feed efficiency, stimulate innate immune responses [29,30,31], and mitigate oxidative stress associated with environmental stressors and antibiotic treatments [23].

However, the biological efficacy of Ulva-derived products depends largely on the extraction technique employed, as this determines both the yield and the phytochemical profile of the recovered bioactive compounds. While dried biomass and conventional solvent extracts have been evaluated as dietary supplements in aquaculture, supercritical fluid extracts remain largely unexplored in fish nutrition.

Supercritical fluid extraction (SFE), most commonly using supercritical carbon dioxide (SC-CO_2_), is regarded as a green extraction technology capable of selectively recovering lipophilic and, in the presence of co-solvents such as ethanol, moderately polar bioactive compounds under relatively mild operating conditions [32,33,34,35,36]. This approach has been successfully applied to various marine macroalgae to obtain carotenoids, phenolic compounds, chlorophylls, fatty acids and sterols [32,37,38,39,40]. In *Ulva lactuca*, SC-CO_2_ extraction has been shown to recover lipophilic compounds, including unsaturated fatty acids, linoleic acid ethyl ester and phytosterols [41], suggesting considerable potential for the development of highly standardized functional feed additives.

Common carp (*Cyprinus carpio*), a widely farmed freshwater species known to be susceptible to management-related stressors including antibiotic administration [22,42,43], was selected as a suitable model for evaluating nutritional strategies against antibiotic-associated physiological disturbance.

Although interest in Ulva-based functional ingredients for aquaculture has grown considerably, most available studies have relied on dried algal biomass or conventional solvent extraction, while the biological effects of phytochemically characterized, solvent-free supercritical fluid extracts remain largely unexplored—particularly regarding their potential to alleviate the physiological disturbances associated with antibiotic administration. To the best of our knowledge, this is the first study to evaluate the effects of a phytochemically characterized *Ulva lactuca* supercritical fluid extract (Ul-SFE) on common carp, both under standard rearing conditions and during administration of two widely used antimicrobial agents, florfenicol and oxytetracycline.

Addressing this gap, and in line with the objectives of the European Green Deal—and specifically its Farm to Fork Strategy, which sets an explicit target of reducing EU sales of antimicrobials for farmed animals and aquaculture by 50% by 2030 (baseline 2018)—the present study aimed to: (i) obtain and phytochemically characterize a supercritical fluid extract of Ulva lactuca (Ul-SFE); (ii) evaluate the effects of its dietary inclusion on the growth performance and physiological status of common carp (*Cyprinus carpio*); (iii) comparatively assess, for the first time in this species, the differential physiological and zootechnical impact of florfenicol and oxytetracycline—two of the most widely used antimicrobials in aquaculture practice—as a relevant model of antibiotic-associated stress; and (iv) determine whether dietary Ul-SFE supplementation can mitigate the physiological and productive costs of antibiotic administration, supporting its potential as a functional feed additive in intensive carp farming.

## 2. Materials and Methods

### 2.1. Raw Material

*Ulva lactuca* biomass was purchased from Algamar company (Pazos de Borbén, Pontevedra, Spain) in 2025 (lot no. 25192-10346). According to the supplier’s label information, these macroalgae were certified as an ecological product (certification ES-ECO-0022-GA) and were manually harvested from the Atlantic coast of northeastern Spain and dried at low temperatures to preserve their nutritional properties for as long as possible.

### 2.2. Chemicals and Reagents

Ethanol >96% was purchased from S.C. Maraton 92 Impex S.R.L. (Bucharest, Romania), and HPLC-grade methanol ≥99.9% was purchased from Honeywell (Seelze, Germany). The n-hexane, acetone, ethyl acetate, acetonitrile, formic acid, and standard compounds as phenolic acids (caffeic acid, protocatechuic acid, vanillic acid, syringic acid, ferulic acid, sinapic acid) and flavonoids (Quercetin and derivatives, catechin and derivatives, hesperidin, luteolin, kaempferol and apigenin) used for HPLC analysis were purchased from Sig-ma-Aldrich (Millipore Sigma, Steinheim, Germany). Methanol was purchased from Chimexim (Bucharest, Romania). All the other reagents were of analytical grade. All the reagents were of analytical grade. Carbon dioxide (99.99% purity) was purchased from Messer Romania (Bucharest, Romania). Helium 6.0, used as a carrier gas, was purchased from SIAD Romania.

### 2.3. Supercritical Carbon Dioxide Extraction of Ulva lactuca

Extractions were carried out using a pilot-plant (Natex, Prozesstechnologie GesmbH, Ternitz, Austria, Fabr. no. 10-023/2011) designed with a cylinder extraction vessel and two separators. Before extraction, the dried Ulva macroalga was ground using a manual grinder (Tristar KM 227, 150 W, Tilburg, The Nederlands). For each batch extraction, 0.220 kg of dried algal biomass was mixed with 7.5% ethanol as co-solvent. Extraction conditions for all batches were based on the optimized parameters established in the experiments of Fabrowska et al. (2016) [32] (pressure of 30 MPa, temperature of 40 °C, and extraction time of 120 min). During extraction, the solvent (CO_2_, 99.99% purity supplied by Messer S.A., Romania) was constantly chilled to remain liquid and recirculated. The solvent was brought to supercritical conditions at 7.38 MPa, and the ABB software indicated a mass flow rate of 21.14 kg/h (ABB—Mannheim, Germany). After extraction, the extracts (Ul-SFE) were collected in vials placed in an ice bath, and the residual ethanol was evaporated using a vacuum rotary evaporator at 40 °C (AVC 2–18, Christ, Shrewsbury, UK). The extraction yield (%*w*/*w*) was gravimetrically calculated as the ratio of the weight of extract (g) and the weight of dry biomass (g). The concentrated extracts were stored at −20 °C until further analyses. Chromatographic and spectrophotometric methods were assessed to identify lipophilic and hydrophilic compounds.

### 2.4. HPLC for Hydrophilic and Lipophilic Compounds

The identification of polyphenolic compounds was performed at wavelengths of 280 nm, and 320 nm according to the method described by Balan et al. (2025) [44]. HPLC analysis was carried out using an Agilent 1200 HPLC system (Agilent Technologies, Santa Clara, CA, USA), equipped with a degasser, a quaternary pump, a column compartment, and a diode-array detector. The BDS Hypersil C18 column (150 mm × 4.6 mm, 5 µm) was used for compound separations under the following conditions: the column temperature was set to 30 °C, the injection volume was 10 µL, and the flow rate was 1 mL/min. The mobile phase consisted of 100% methanol (solvent A) and 10% formic acid (solvent B), with the following gradient program: 0–20 min, 9% A/91% B; 20–30 min, 35% A/65% B; 30–40 min, 50% A/50% B; 40–45 min, 9% A/91% B. Data acquisition and processing were performed automatically using Agilent ChemStation software (Rev. B.04.03), based on calibration curves obtained with standard compounds. Quantification of the identified polyphenolic compounds was performed using calibration curves established with HPLC analytical standards of the corresponding compounds. All measurements were conducted in triplicate. The results were expressed ng/g d.w. alga.

The identification of lipophilic compounds, particularly carotenoids, was performed at 450 nm, a wavelength commonly employed and reported in previous studies for carotenoid detection [45,46,47]. Results were expressed as µg/g d.w. extract after 30 min of elution and data acquisition, accounting for the calibration curves for each identified compound. Data acquisition and processing were performed automatically using Agilent ChemStation software (Rev. B.04.03), based on calibration curves obtained with standard compounds. All measurements were conducted in triplicate.

### 2.5. GC-MS Analysis of the Ul-SFE Extract

GC-MS Clarus 680/SQ8T (Perkin Elmer, Waltham, MA, USA) equipped with an Elite-5MS capillary column (30 m × 0.25 mm i.d., 0.25 µm film thickness (Perkin Elmer, Waltham, MA, USA), using helium as carrier gas (flow rate of 1.0 mL/min) was used to perform the GC-MS analysis of the *Ul*-SFE extract. Briefly, 0.1 g of extract was diluted with methanol at a final volume of 10 mL. The solution obtained was injected at 1 µL in split mode (split ratio 20:1), and the temperature program was as follows: initial temperature: 50 °C (hold time 2 min), increase by 10 °C/min to 310 °C, hold time 4 min. The injector temperature was set to 220 °C. The MS operating conditions were: source temperature 300 °C, transfer line temperature 220 °C, electron impact ionization EI+ at 70 eV, and a solvent delay of 3 min. MS detector was operated to acquire all the *m*/*z* ions from 35 to 600. For qualitative purposes, the NIST/ MS search library (version V2.4) and Wiley 9 were used, and a matching criterion of 80% was applied in order to select the possible hits.

### 2.6. Experimental Protocol and Fish Feeding Regime

The present experiment was conducted in the experimental unit of the Pilot Station of Aquaculture of the Faculty of Food Science and Engineering, University “Dunărea de Jos” of Galati, Romania. The experimental set-up for the treatment groups was carried out in recirculating systems comprising either 6 tanks of 500 L (Phase 1) or 18 glass aquaria of 130 L (Phase 2), under standardized laboratory conditions. The systems were equipped with water filtration systems, ensuring identical growth conditions for experimental groups whin each trial.

A total of 500 common carp (*Cyprinus carpio*) juveniles were supplied by a local fish farm, selected based on geographic proximity to minimize transport-related stress, with fish of homogeneous size specifically requested from the supplier. Upon arrival, fish were held in quarantine for seven days for health evaluation, during which no clinical signs of disease were observed and no treatments were applied. Following quarantine, 360 fish (mean initial weight 20.20 ± 0.13 g) were selected by excluding individuals outside the target weight range, ensuring a homogeneous initial population, and acclimatized to experimental conditions for a further seven days. During acclimatization, fish were fed a commercial extruded diet (Coppens Premium Select 2.0, Coppens International, Nijkerk, The Netherlands; Supplementary Table S1). After acclimatization, fish were randomly allocated to two dietary treatments (conventional commercial diet–C; the same diet supplemented with Ul-SFE extract–U; n = 180 each, 3 tanks/treatment, 60 fish/tank). The Ul-SFE dose (50 mg kg^−1^ feed) was selected based on biologically effective concentrations reported for other Ulva species in different fish species [31,48], as no data exist for supercritical Ulva extracts specifically. Feed was prepared weekly: both diets underwent identical gelatin-coating (5%, *w*/*w*) and oven-drying (40 °C), with extract added only to the U coating solution, allowing the Control diet to serve as a vehicle control.

In Phase 2, fish from each dietary treatment were randomly redistributed into three sub-treatments—no antibiotic, oxytetracycline (OTC), or florfenicol (FF)—yielding six experimental groups (C, C + OTC, C + FF, U, U + OTC, U + FF; n = 60/group, 3 aquariums × 20 fish/aquarium; Supplementary Table S2). Antibiotic doses (15 mg kg^−1^ body weight day^−1^ FF, Florfenidem 50, 500 mg/g florfenicol, Delos; 75 mg kg^−1^ body weight day^−1^ OTC, Oxytetracycline FP 90%, 900 mg/g oxytetracycline hydrochloride, Farmavet; both from Biotur Exim SRL, Alexandria, Romania) corresponded to standard aquaculture practice [18,49]. All six diets were prepared simultaneously using the same gelatin-coating/oven-drying procedure as Phase 1, with gelatin alone (C), gelatin + extract (U), gelatin + antibiotic (C + FF, C + OTC), or gelatin + extract + antibiotic (U + FF, U + OTC) as appropriate.

Fish were fed twice daily at 2% body weight, with feed and antibiotic doses adjusted periodically to tank biomass. The full daily antibiotic dose was incorporated into the first meal only, ensuring complete ingestion (fully consumed in all tanks throughout the trial); the second, non-medicated meal was monitored, with uneaten feed collected, weighed, and used to calculate actual intake. Survival was assessed daily. Water quality parameters were monitored daily throughout the experiment and maintained within optimal limits for common carp: water temperature 20.10 ± 1.2 °C, pH 7.68 ± 0.20, dissolved oxygen ≥ 7 mg/L, and nitrogen compounds (N-NO_3_^−^, N-NO_2_^−^, N-NH_4_^+^) within safe limits.

### 2.7. Evaluation of Growth Performance

Growth performance was assessed at the end of each experimental stage and periodically (P1–P4) for the first stage. At each sampling point, fish from each tank were bulk weighed to determine total biomass, and a representative subsample was weighed to calculate mean body weight. Total feed intake (TFI) was recorded daily, and cumulative feed consumption per tank was used for subsequent calculation of feed utilization indices (Section 2.9). All measurements were performed on a per-tank basis, considering each tank as the experimental unit.

### 2.8. Blood Sampling and Physiological Assessments

After second stage of the experiment, five fish from each experimental variant were randomly selected for hematological assessment. To minimize handling-related stress, individuals were anesthetized in a 2-phenoxyethanol solution (0.7 mL/L) until a deep anesthetic stage was achieved. This anesthetic agent was chosen due to its minimal interference with hematological parameters. Blood collection was rapidly performed by puncturing the caudal vein with heparinized syringes, after which samples were transferred into sterile collection tubes and maintained on ice during transport to the laboratory.

For biochemical determinations, blood samples were centrifuged at 3500 rpm (1166× *g*) for 10 min using a Hettich Mikro 120 centrifuge (Hettich Zentrifugen, Tuttlingen, Germany), and the resulting plasma was carefully separated into 1.5 mL Eppendorf tubes. Plasma biochemical parameters were analyzed with a VetTest^®^ Chemistry Analyzer and corresponding IDEXX VetTest reagent kits (IDEXX Laboratories, Inc., Westbrook, ME, USA). Lipid peroxidation status was assessed by quantifying malondialdehyde (MDA, nmol/mL) levels using the method described by Ohkawa (1979) [50]. The absorbance of the reaction mixtures was subsequently measured spectrophotometrically at 532 nm (SPECORD 210 spectrophotometer, Analytik Jena AG, Jena, Germany). Total antioxidant capacity (TAC, mMol Trolox equivalents) was evaluated spectrophotometrically using the ABTS radical cation decolorization assay (2,2′-azinobis-(3-ethylbenzothiazoline-6-sulfonic acid)), with absorbance readings performed at 734 nm, following the methodology previously described by [51].

Plasma lysozyme activity was assessed using a turbidimetric procedure based on the Enzymatic Activity of Lysozyme Protocol (Sigma, EC 3.2.1.17, Sigma-Aldrich, St. Louis, MO, USA). Briefly, a 66 mM potassium phosphate buffer (pH 6.24 at 25 °C) was combined with a 0.01% (*w*/*v*) suspension of *Micrococcus lysodeikticus* (Sigma, M3770, Sigma-Aldrich, St. Louis, MO, USA). Lyophilized chicken egg white lysozyme (Sigma, L6876, Sigma-Aldrich, St. Louis, MO, USA) served as the calibration standard. Lysozyme activity was expressed as the amount of enzyme causing a decrease in absorbance of 0.001 per minute at 450 nm.

### 2.9. Analysis of the Experimental Data

Growth and feed utilization parameters were calculated using tank-level data, considering each tank as the experimental unit. The following performance indicators were determined:

Total weight gain (TWG, g) = Final biomass (g) − Initial biomass (g)

Individual weight gain (IWG, g) = Final mean body weight (g) − Initial mean body weight (g)

Feed conversion ratio (FCR) = Feed intake (g)/Biomass gain (g)

Specific growth rate (SGR, % day^−1^) = 100 × [ln(Final body weight) − ln(Initial body weight)]/number of rearing days

Protein efficiency ratio (PER) = Biomass gain (g)/Protein intake (g)

Survival (%) = (Final fish number/Initial fish number) × 100

Data obtained from growth performance, biochemical, oxidative stress, and immune-related analyses were statistically processed using IBM SPSS Statistics software (version 20, IBM Corp., Armonk, NY, USA). Prior to analysis, data distribution normality and homogeneity of variances were verified using the Shapiro–Wilk and Levene tests, respectively. Growth performance parameters recorded in Phase 1, during the different experimental stages (P1–P4) were analyzed using a repeated-measures ANOVA in a mixed (split-plot) design, with dietary treatment (Control vs. Ul-SFE) as the between-subject factor and growth period as the within-subject repeated factor, and replicate tank as the experimental unit. Mauchly’s test was used to verify the assumption of sphericity; as this assumption was met for all three indicators (*p* > 0.05), results are reported without correction. For data obtained in Phase 2 (both growth performance indicators and biochemical markers), differences among experimental groups were evaluated by two-way analysis of variance (Two-way ANOVA) procedure to assess the individual effects of Ul-SFE supplemented diet and therapeutic-dose antibiotic administration, as well as their interaction (Diet × Antibiotic). When significant differences were detected, Tukey’s HSD post hoc test was applied for pairwise comparisons among means. Results are presented as mean ± standard deviation (SD), and differences were considered statistically significant at *p* < 0.05. Extreme outlier values and missing data identified during preliminary screening were excluded from the statistical analysis only for the affected parameters.

To obtain an integrated overview of the physiological responses induced by dietary supplementation and antibiotic administration, a principal component analysis (PCA) was performed using standardized biomarker values (z-score transformation). Because several measured variables described the same physiological process and exhibited strong pairwise correlations (e.g., total protein, globulins, and albumin/globulin ratio), a functional biomarker approach was adopted to reduce redundancy and improve biological interpretability of the ordination. Consequently, the PCA included representative biomarkers describing protein status (TP, ALB), hepatic function (ALT, TBIL), digestive activity (AMYL), energy metabolism (GLU, CHOL), mineral homeostasis (Ca), oxidative status (MDA, TAC), and innate immunity (LYZ). Principal components with the highest explained variance were retained for graphical representation. Individual fish were projected onto the first two principal components, and 95% confidence ellipses were calculated for each experimental group to visualize group dispersion and overlap. Principal Component Analysis (PCA) was performed using Python software (version 3.11) with the libraries scikit-learn for multivariate analysis and Matplotlib (version 3.10.8) for graphical representation.

## 3. Results

### 3.1. HPLC Analysis of the Ul-SFE Extract

In our experiment, the extraction process yielded 0.5% (*w*/*w*) dry extract. The *Ul* –SFE extract was subjected to HPLC analysis to establish the bioactive compound profile. Therefore, 14 bioactive compounds were identified, among which 6 were phenolic acids (vanillic acid, protocatechuic acid, caffeic acid, syringic acid, ferulic acid, synaptic acid), and 8 were flavonoids (epigallocatechin, epicatechin galate, hesperidin, quercetin 3-diglucozide, quercetin, keempherol, apigenin) (Table 1, Supplementary Figure S1).

### 3.2. GC-MS Analysis of the Ul-SFE Extract

Green extraction methods are used for environmental concerns and minimize or eliminate the use of organic solvents [70]. SC-CO_2_ is a nonpolar solvent with higher selectivity for the lipophilic compounds. For our *Ul*-SFE extract, the GC-MS chromatogram revealed the predominance of the lipids, pigments, and sterols, which were qualitatively identified (Table 2, Supplementary Figure S2). The extract contains, as a major compound (21.23%), phytol known for its antioxidant, antimicrobial, and anti-inflammatory properties [71,72,73]. The highest concentration of phytol demonstrated that the extraction conditions successfully damaged the alga cell wall, and degraded the chloroplast during the high-pressure extraction. Therefore, phytol showed characteristic fragment ions, typical of an acyclic diterpene alcohol that forms a lipophilic chain of chlorophyll molecules from the algae. The probability value of 91.50%, demonstrates the accuracy of the identification, while library NIST matches values for Kovats Retention Indices as 2110–2140.

In our extract was identified 8-Heptadecane (6.89% and RT:14.50 min), known for antimicrobial and antifungal effect, but also as a volatile algae aroma compound [74]. His probability to identification was higher than 80% and the RIL value was 1700. Moreover, lower individual area peak (<2%) was calculated for squalene, and sesquiterpene esters that presented a probability of identification around of 75%. The squalene (0.71%; RT: 24.37 min, *m*/*z* 410) being a high molecular-weight compound eluted in the final phase of the GC-MS analysis, present a probability of identification 77.40%. It is noticed that squalene is a biochemical precursor for sterol biosynthesis in macroalgae [83,84], so its presence in *Ul*-SFE extract was confirmed by the RIL of 2800–2850, values in the same range as sterols.

Additionally, other compounds were tentatively identified on the basis of the NIST library and *m*/*z* spectra and included in the category of sterol isomers or terpenoid esters, even they did not show a probability of identification higher than 80%.

### 3.3. Growth Performance and Feed Utilization

Growth performance indicators (FCR, SGR, PER) for carp fed the Control and Ul-SFE-supplemented diets across the four experimental periods (P1–P4) are presented in Table 3. Results confirmed a statistically significant overall effect of dietary treatment on FCR, SGR, and PER. The diet × period interaction was not significant for any of the three indicators (*p* > 0.5).

Growth performance and feed utilization indices of common carp fed the control (C) and *Ul*-SFE-supplemented diets (U), with or without antibiotic co-administration (florfenicol, FF; oxytetracycline, OTC), over the 10-day post-treatment period are summarized in Table 4. The U groups achieved the best overall performance, with the most efficient FCR (1.51 ± 0.06), and the highest SGR (1.80 ± 0.06%/day), while florfenicol administration was associated with the poorest feed efficiency in both dietary backgrounds (highest FCR, lowest SGR). The diet × antibiotic interaction was significant only for TFI (*p* = 0.002), indicating that feed intake responded differently to antibiotic administration depending on the dietary background; for all other parameters, diet and antibiotic effects acted independently of one another. Survival remained high across all groups (90–100%), with the lowest value recorded in the C + FF groups.

### 3.4. Biochemical Indicators of Metabolic Responses

Dietary supplementation with *Ul*-SFE extract significantly increased total protein (TP), albumin (ALB), globulin (GLOB), and phosphorus (P) compared with the conventional diet. Antibiotic administration significantly affected TP, ALB, the A/G ratio, ALT, BUN (Blood Urea Nitrogen), amylase (AMYL), glucose (GLU), cholesterol (CHOL), calcium (Ca), and bilirubin (TBIL). Notably, *Ul*-SFE extract supplementation significantly attenuated several antibiotic-induced changes, particularly by restoring TP, ALB, GLOB, A/G ratio, TBIL, ALT, BUN, GLU and CHOL toward control values (Table 5).

Two-way ANOVA revealed that the measured biochemical biomarkers were differentially influenced by dietary supplementation with *Ul*-SFE extract and antibiotic administration. The most pronounced effects were associated with the antibiotic factor. In contrast, dietary supplementation had significant effects on fewer variables, and the interaction between the two factors was generally limited (Table 6).

### 3.5. Oxidative Stress and Innate Immune Response

Oxidative stress and immune-related parameters were influenced by both antibiotic and *Ul*-SFE extract supplementation (Table 7). Lysozyme activity was highest in the U groups, while antibiotic-treated groups exhibited significantly lower lysozyme values. MDA levels, an indicator of lipid peroxidation, increased significantly in fish exposed to antibiotics, particularly in the C + OTC group, indicating enhanced oxidative stress. However, the inclusion of *Ul*-SFE extract significantly reduced MDA concentrations in both antibiotic-treated variants. TAC values showed a decreasing trend under antibiotic exposure, especially in OTC-treated fish, while UE supplementation alone maintained the highest antioxidant capacity.

**Figure 1:**
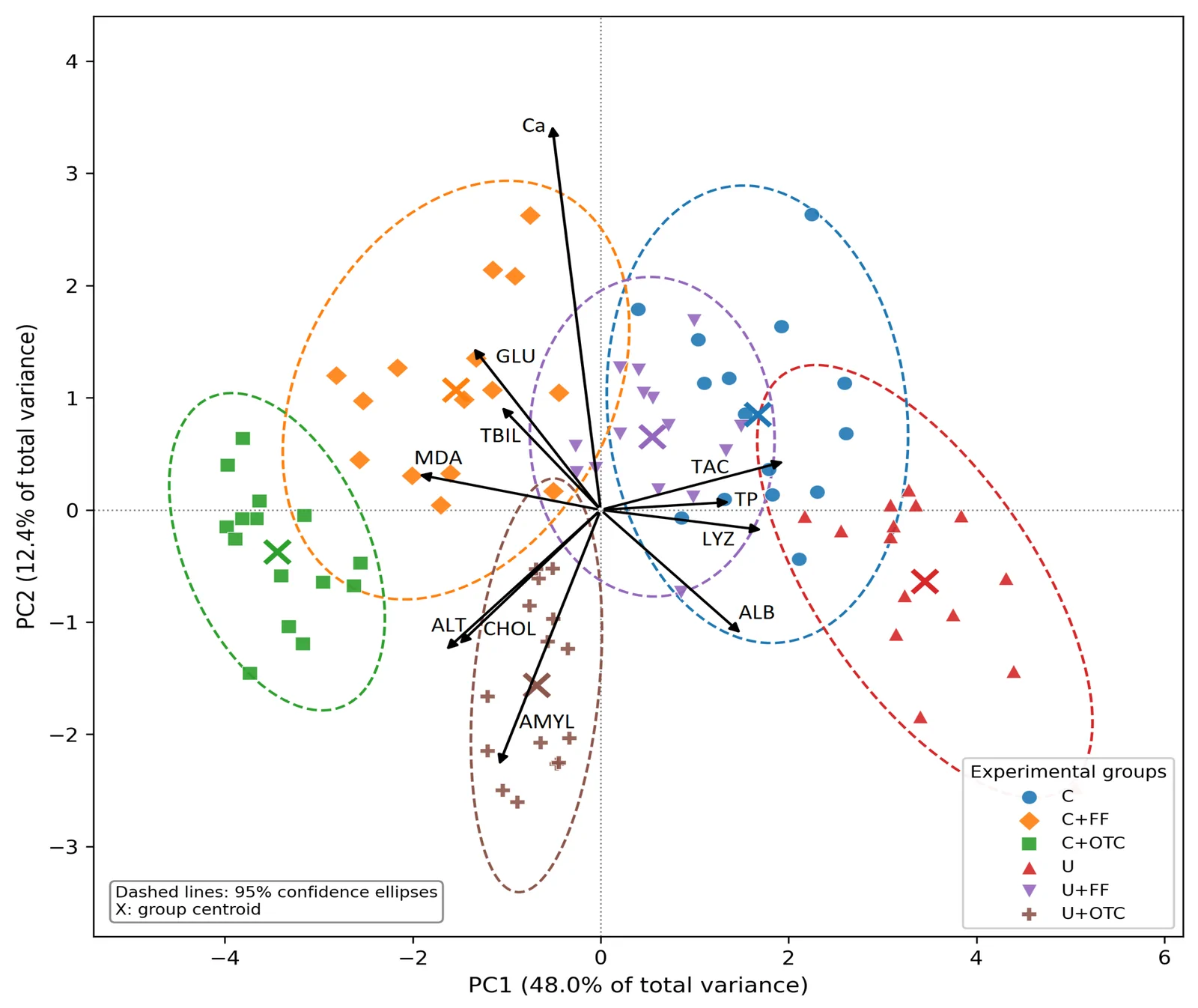
Principal component analysis (PCA) biplot of functional biomarkers.

To obtain an integrated overview of the physiological responses elicited by dietary *Ul*-SFE supplementation and antibiotic administration, a principal component analysis (PCA) was conducted using the selected functional biomarkers. The first two principal components accounted for 60.43% of the total variance (PC1 = 47.99%; PC2 = 12.43%). PC1 was predominantly driven by oxidative stress and antioxidant biomarkers together with indicators of hepatic and immune status, whereas PC2 was mainly influenced by biomarkers related to digestive function and mineral metabolism (Figure 1).

## 4. Discussion

Antibiotics are widely used in aquaculture to control bacterial diseases; however, their extensive use and misuse contribute to the development of antimicrobial resistance and may exert adverse effects on aquatic ecosystems and fish physiology. Beyond their antimicrobial activity, antibiotics are known to disrupt microbial communities and induce physiological alterations in fish, including changes in immune function and oxidative stress responses [14,42,85] or cytotoxic and genotoxic effects [86]. Accordingly, the present study was designed not only to evaluate the effects of dietary supplementation with a phytochemically characterized *Ulva lactuca* supercritical fluid extract (Ul-SFE) on the growth performance and physiological status of common carp under normal rearing conditions, but also to investigate whether this dietary strategy could modulate physiological and oxidative stress responses during therapeutic administration of florfenicol and oxytetracycline.

### 4.1. Ul-SFE Extract Characterization

In the present study, supercritical carbon dioxide extraction (SFE-CO_2_) was conducted to isolate bioactive compounds from commercial dried *Ulva lactuca*. In supercritical conditions, the carbon dioxide is above its critical pressure and temperature, resulting in easier diffusion through solid materials and therefore faster extraction yields [34]. Usually, supercritical carbon dioxide (SC-CO_2_) is used to extract non-polar compounds and, in the presence of a co-solvent such as ethanol, extraction of the medium-polarity natural bioactive compounds is possible [32,87]. In the case of marine macroalgal species, SC-CO_2_ has been used previously for the extraction of carotenoids [38,39,40] phenolic compounds [37], chlorophylls [32], and lipophilic compounds [41].

In our experiment, the extraction process yielded lower compared with other methods. For instance, other authors [80] reported up to 9.45% for the maceration extraction, and 1.18% for the methanolic extraction of Ulva lactuca collected from Nador lagoon, Moroc. However, our results are in accordance with studies involving SFE-CO_2_ extraction. For instance, for Ulva flexuosa, extracted under SFE-CO_2_ conditions (at 30 MPa, 40 °C, and 11.4% *w*/*v* ethanol added as cosolvent) a yield of 0.4% has been previously reported [32].

A lower yield as 0.19% (*w*/*w*) was also reported by other authors [41] for SC-CO_2_ extraction of Ulva lactuca collected from the coastal regions of Rameswaram and Mandapam, India. The differences in the yield values are motivated by the parameters involved in the SC-CO_2_ process and by biomass properties.

In general, SC-CO_2_ enables the extraction of a wide range of bioactive compounds from dried microalgae, generally of low molecular weight [55]. These include phenolic compounds, carotenoids, terpenes (mono- and diterpenes), sterols, fatty acids, proteins, and carbohydrates [56,70,88].

In our study HPLC analysis demonstrated that the *Ul*-SFE extract was particularly rich in flavan-3-ols, with epigallocatechin and epicatechin gallate representing the predominant phenolic constituents, followed by flavonols (kaempferol, quercetin and quercetin 3-diglucoside), flavones (luteolin and apigenin) and several phenolic acids, including protocatechuic, caffeic and vanillic acids. These compounds are widely recognized as potent antioxidants capable of scavenging reactive oxygen species (ROS), inhibiting lipid peroxidation, regulating inflammatory pathways and enhancing endogenous antioxidant defense mechanisms (Table 1).

In parallel, GC-MS analysis identified a lipophilic fraction dominated by phytol, which accounted for more than 21% of the total chromatographic area, together with several biologically active terpenoids, including manool, sesquiterpene esters, squalene, methyl palmitate, phytosterol derivatives and 2-oleyloxy-1-ethanol. These metabolites have been associated with antioxidant, anti-inflammatory, antimicrobial, membrane-protective and immunomodulatory activities (Table 2). In particular, phytol and squalene are well known for stabilizing cellular membranes and protecting lipid-rich structures against oxidative damage, whereas terpenoid compounds such as manool contribute to the modulation of inflammatory responses and cellular signaling.

### 4.2. Growth Performance Assessment

Growth performance represents an integrative indicator of fish health and nutritional efficiency; therefore, considering the antioxidant and metabolic regulatory properties of the bioactive compounds identified in Ul-SFE, beneficial effects on growth-related parameters were expected. Across the four-period trial, both diets showed the expected progression in growth performance, with FCR, SGR, and PER all improving markedly from P3 onward. Because the daily feed allowance was adjusted at each weighing to maintain a constant feeding rate relative to biomass, this improvement is unlikely to reflect a change in feeding regimen and more plausibly reflects normal ontogenetic changes in metabolic and digestive efficiency as the fish grew. Statistical analysis showed that dietary treatment had a significant overall effect on FCR, SGR, and PER (*p* < 0.05 for all three indicators), with the Ul-SFE-supplemented diet outperforming the control diet. Notably, the diet × period interaction was not significant for any indicator, suggesting that this advantage was consistent throughout the trial rather than emerging progressively over time. Thus, dietary supplementation with *Ul*-SFE reduced FCR from 1.48 to 1.37, representing an improvement of approximately 7%. Although numerically modest, such a reduction may have important practical implications in commercial aquaculture by decreasing feed consumption and production costs, given that feed commonly accounts for more than 50% of total operating expenses [89].

The results obtained in the present study are consistent with previous reports indicating that macroalgal supplements primarily enhance fish health and physiological status, whereas their effects on growth performance are often modest. For example, some authors reported positive, albeit non-significant, improvements in the growth performance of *Nile tilapia* supplemented with *Ulva fasciata* extract [48] while a dietary mixture of *Ulva lactuca*, *Jania rubens*, and *Pterocladia capillacea* significantly improved both growth performance and antioxidant status in striped catfish [29].

The beneficial effects of macroalgae are highly dependent on the algal species, processing method, and dietary inclusion level. High levels of macroalgal biomass in aquafeeds may impair nutrient digestibility due to the elevated content of structural polysaccharides, fiber, and antinutritional compounds. In contrast, moderate supplementation, particularly through concentrated extracts rather than whole algal biomass, generally enhances physiological performance via the action of bioactive metabolites, rather than by substantially increasing the nutritional value of the diet itself [90,91,92].

In the subsequent antibiotic-challenge phase, diet had a significant main effect on growth performance indicators (Table 4), antibiotic administration reducing TWG by approximately 10.6% (C + FF) and 7.8% (C + OTC) relative to the non-medicated control, with florfenicol producing the larger reduction in the two antibiotics. Dietary Ul-SFE supplementation partially counteracted these reductions, limiting the TWG decrease to approximately 5.5% in Ul-SFE + FF and to only 2.9% in Ul-SFE + OTC relative to the non-medicated Ul-SFE group. A significant diet × antibiotic interaction was observed for TFI, indicating that feed intake responded differently to antibiotic administration depending on dietary background: intake declined markedly following antibiotic administration in the Control groups but remained comparatively stable in the Ul-SFE-supplemented groups. Antibiotic exposure significantly increased FCR and reduced SGR and PER (main effects, with no significant diet × antibiotic interaction), while Ul-SFE supplementation consistently shifted these technological indicators toward more favorable values, independently of the antibiotic administered. The findings of the present study are consistent with previous reports involving different algal species. The combined use of algal-based feed additives and antibiotics has been shown to support fish health, despite the physiological stress typically associated with antimicrobial treatments. For instance, has been demonstrated that co-administration of *Spirulina platensis* with florfenicol improved physiological status in tilapia [93], while supplementation with *Chlorella vulgaris* has been shown to enhance growth performance and survival in pathogen-challenged fish, suggesting that algal bioactive compounds may promote nutrient assimilation and metabolic efficiency under stress conditions [94].

Growth depression is widely recognized as a tertiary stress response resulting from the cumulative energetic costs of maintaining antioxidant defenses, immune activation and metabolic homeostasis [95]. Therefore, the ability of *Ul*-SFE to preserve growth performance under antibiotic challenge is likely a consequence of its antioxidant and immunomodulatory properties, which reduced the need to divert metabolic resources from growth toward physiological maintenance [96].

### 4.3. Carbohydrate and Protein Metabolism

Beyond growth performance, the evaluation of biochemical, oxidative stress, and immune-related biomarkers provides a deeper understanding of the physiological consequences associated with antibiotic exposure and the potential protective role of *Ul*-SFE supplementation.

Among biochemical markers, glucose is a well-established indicator of the secondary stress response in fish, reflecting metabolic adjustments mediated by cortisol and enhanced gluconeogenesis [97,98]. In our study, glucose levels in the control group were within the range reported for common carp under both farm and experimental conditions [99,100]. However, under antibiotic exposure, glucose in the present study behaved primarily as a stress-related parameter, increasing in both antibiotic-treated fish groups, especially in OTC groups. Hyperglycemia is widely recognized as a typical response to chemical stress in fish, reflecting activation of the hypothalamic-pituitary-interrenal (HPI) axis and subsequent cortisol-mediated gluconeogenesis [101]. However, responses appear to be species- and context-dependent. In common carp, Kondera et al. [102] reported minimal disruption of glucose levels following short-term oral administration of therapeutic OTC, suggesting that acute exposure may have limited metabolic consequences in healthy fish.

In contrast, studies on other species indicate a more pronounced effect. In Nile tilapia (*Oreochromis niloticus*), oral administration of OTC at 80–800 mg/kg biomass/day for 10 days resulted in significant increases in serum glucose and other stress-related biomarkers, with incomplete recovery following withdrawal [103]. Similarly, florfenicol exposure in tilapia at 15 and 45 mg/kg biomass/day for 10 days induced a dose-dependent increase in glucose levels, with prolonged elevation at higher concentrations [104]. These findings suggest that while short-term therapeutic OTC may induce limited metabolic disturbance in carp, prolonged or high-dose exposure to both OTC and FF is generally associated with hyperglycemia as part of a generalized stress response.

Regarding *Ul*-SFE supplementation, our results indicate a stabilizing effect on glucose metabolism. In antibiotic-treated groups receiving *Ul*-SFE, the increase in glucose was attenuated compared to fish receiving medicated feed alone, suggesting partial mitigation of stress-induced metabolic disturbances. Similar findings have been reported in gibel carp (*Carassius gibelio*), where supplementation with macroalgal extracts based on *Ulva lactuca* and *Solieria chordal* is did not significantly alter plasma glucose levels, indicating metabolic safety under routine feeding conditions [105].

Evidence from microalgal studies further supports the protective role of algal-derived compounds. For instance, dietary *Spirulina platensis* has been shown to alleviate florfenicol-induced physiological disturbances in Nile tilapia, primarily through antioxidant and hepatoprotective mechanisms, although effects on glucose were less pronounced [93,106].

Amylase activity increased significantly under antibiotic treatment, suggesting metabolic disturbances associated with chemotherapeutic stress, possibly linked to altered carbohydrate metabolism and hepatopancreatic functional responses. This may also represent a compensatory response to disrupted nutrient absorption or microbiota imbalance. Notably, a significant Diet × Antibiotic interaction (*p* < 0.001) indicated that this response was also diet-dependent, with Ulva supplementation alone already elevating amylase activity to levels comparable with the C + FF group, while its combination with antibiotics did not produce a further additive increase. Similar increases in amylase activity have been reported under both dietary and environmental stress conditions [107].

Total protein (TP) and globulin (GLOB) fractions were also affected by antibiotic exposure, particularly under OTC treatment. The decrease in total protein and globulin levels, together with the increase in the albumin-to-globulin ratio (A/G), may indicate impaired hepatic protein synthesis and suppression of humoral immune function, as globulins are closely associated with immunocompetence in fish [108,109]. The pattern found in the present study is consistent with findings reported by other authors. For instance, juvenile common carp exposed to a sub-lethal concentration of oxytetracycline (80 mg/L) for 24, 48, 72, and 96 h exhibited a progressive and significant decline in total plasma protein levels over time, suggesting an inhibition of protein synthesis and disturbances in metabolic homeostasis [110]. Similarly, in *Oreochromis niloticus* fed 100 mg/kg OTC for 12 weeks, significant reductions in immunoglobulins were associated with physiological stress and immunosuppression [111]. Compared with oxytetracycline, florfenicol (FF) produced milder effects, maintaining TP values closer to the control and slightly increasing globulin levels, suggesting a more balanced physiological response. This aligns with studies indicating that FF has a lower disruptive impact on protein metabolism compared to tetracyclines, especially when administered at therapeutic levels [18].

In the present study, dietary *Ul*-SFE significantly improved protein status. Moreover, the normalization of the A/G ratio in U + FF and U + OTC groups indicates the potential of *Ul*-SFE to restore the balance between metabolic and immune protein fractions. Comparable responses have also been reported in *Oreochromis niloticus* fed diets supplemented with 50 mg/kg *Ulva fasciata* extract, where improvements in protein metabolism, immune responsiveness, and nutrient utilization efficiency were observed [112]. These beneficial effects of macroalgae-based diets are generally associated with the presence of polyphenols, sulfated polysaccharides, and other bioactive compounds capable of modulating immune pathways, enhancing digestive efficiency, and promoting nitrogen retention and protein synthesis [113,114].

### 4.4. Liver and Kidney Function

Hepatic stress associated with antibiotic exposure was reflected by increased Alkaline phosphatase (ALP) and alanine aminotransferase (ALT) activity along with elevated cholesterol (CHOL) levels. However, only ALT was significantly higher in the FF and OTC groups, compared to the control, indicating hepatocellular damage. This observation is consistent with previous studies reporting elevated ALT activity and histopathological alterations in the liver of *Oreochromis niloticus* exposed to therapeutic doses of oxytetracycline, effects that were associated with oxidative stress, hepatocellular injury, and mitochondrial dysfunction [111,115]. TBIL remained overall low (0.20–0.26 mg/dL), and although a slight increase was observed in the antibiotic-treated groups, the concentrations remained within the physiological range generally considered normal for *Cyprinus carpio* [116]. This finding is consistent with fish physiology, in which bilirubin metabolism differs substantially from that of mammals and circulating bilirubin levels are typically minimal under healthy conditions. Nevertheless, increases in TBIL may still reflect early hepatic disturbances, as hyperbilirubinemia in fish has been associated with hematotoxic effects, hemolytic processes, bilirubin overproduction, or hepatotoxicity leading to impaired bilirubin metabolism and detoxification pathways [117]. Florfenicol, induced moderate increases in ALT, confirming a milder hepatic impact. These results are consistent with studies showing reversible hepatic changes under therapeutic FF exposure [118].

*Ul*-SFE reduced ALT and CHOL values in antibiotic-treated groups, confirming a hepatoprotective effect, maintaining the hepatic enzymes close to the control groups. This has been widely attributed to the antioxidant properties of macroalgal compounds, which reduce oxidative stress [114] and stabilize hepatocyte membranes [29]. Similar improvements in liver functional status following macroalgal supplementation have been reported in several fish species. Thus, dietary *Ulva intestinalis* improved total protein, immunoglobulin levels, and immune-related gene expression in common carp without inducing increases in ALT, AST, or ALP activities, suggesting the absence of hepatic stress [119]. Likewise, in yellow catfish fed with *Sargassum horneri*, enhanced hepatic antioxidant capacity, increased serum protein levels, and reduced lipid peroxidation were observed [120], while rainbow trout supplemented with *Sargassum* and *Gracilaria* had stable hepatic enzyme activities and improved metabolic profiles [121].

Kidney health is an important component of antibiotic safety evaluation in aquaculture because the kidneys are involved in osmoregulation, nitrogen excretion, and xenobiotic elimination. Exposure to OTC and FF induce renal dysfunction associated with oxidative and metabolic stress [102]. In general, blood urea nitrogen (BUN) and creatinine (CREA) are commonly used indicators of renal and metabolic status in fish [122] representing also a glomerular filtration rate index.

In the present study, BUN showed a significant increase following antibiotic exposure, although values remaining below the reference range reported for *Cyprinus carpio* [99]. Because teleost fish excrete nitrogen mainly as ammonia, BUN variations are considered more closely related to protein catabolism and nitrogen metabolism than to severe renal dysfunction [123]. In parallel, creatinine concentrations showed slight, non-significant elevation following antibiotic exposure suggesting possible renal physiological stress [124]. Similar increases in CREA levels have been reported in *Oncorhynchus mykiss* and *Oreochromis niloticus* exposed to therapeutic or elevated doses of OTC [103,125], as well as in Nile tilapia treated with elevated FF concentrations [126]. However, contradictory findings have also been documented; no significant impairment of renal function was reported in Nile tilapia following OTC administration [110]. Therefore, the nephrotoxic effects of antibiotics may vary depending on species, dosage, exposure duration, and experimental conditions.

In the present study, dietary supplementation with *Ul*-SFE did not significantly affect BUN and CREA levels; however, a significant BUN reduction was observed in fish receiving both *Ul*-SFE and medicated feed compared to those treated exclusively with antibiotics. The present findings support previous evidence indicating that *Ulva lactuca*-derived bioactive compounds may exert ameliorative effects against oxidative stress–related renal dysfunction [127].

In the present study, plasma calcium and phosphorus were both significantly influenced by diet and antibiotic treatment. Notably, the groups with the lowest calcium values (U and U + OTC) also showed the highest phosphorus values. This suggests that dietary Ulva supplementation may influence calcium and phosphorus levels in an interrelated manner, an effect that appears to depend on the co-administered antibiotic. The observed increase in plasma calcium in FF-treated groups may reflect a cortisol-mediated stress response, known to enhance branchial calcium uptake in teleosts [128], a mechanism that is pharmacologically distinct from the calcium-chelating properties of tetracyclines such as OTC. Fish receiving the *Ul*-SFE diet exhibited a more stable mineral profile, which may be associated with the naturally high mineral content of Ulva spp., including calcium and phosphorus, as well as their potential to improve nutrient digestibility and mineral utilization efficiency [129].

### 4.5. Oxidative Stress

In the present trial, the administration of both antibiotics induced evident oxidative and immune-related alterations in *Cyprinus carpio*, although the magnitude of the response differed between OTC and FF treatments. Oxytetracycline appeared to exert a stronger pro-oxidative effect, as reflected by the more pronounced increase in lipid peroxidation and the greater depletion of antioxidant capacity compared to florfenicol. At the same time, the reduction in lysozyme activity observed in antibiotic-treated fish suggests a partial suppression of innate immune responsiveness under chemotherapeutic stress. These findings are consistent with previous studies reporting that OTC and FF can disrupt oxidative balance and modulate immune function in fish, although the direction and intensity of the response remain highly species- and dose-dependent [20,130,131]. In *Cyprinus carpio*, elevated MDA levels accompanied by impaired antioxidant defenses in the liver and kidney have been reported following exposure to high OTC doses (150–300 mg/kg), with oxidative disturbances persisting even during the withdrawal period [132]. Comparable results were obtained in stinging catfish [131], pearl gentian grouper [14] and Nile tilapia during the oral supplementation of OTC at therapeutic doses [133] or environmental doses [134]. In comparison with OTC, FF has been associated with milder alterations in previous studies [18], although high doses may still impair antioxidant and immune-related pathways [135]. Nevertheless, contradictory findings are also reported. For instance, florfenicol was shown to enhance lysozyme activity in the blood of electric yellow cichlid fed *Ulva intestinalis* and *Gracilariopsis persica* [136] and stimulate immune-related gene expression in zebrafish, suggesting a response characterized by initial immune activation followed by suppression during prolonged exposure [16]. Similarly, using various doses of OTC and FF on the SHK-11 cell line of *Salmo salar*, other authors demonstrated that both OTC and FF initially stimulated antioxidant pathways before inducing oxidative exhaustion during extended exposure [20]. Such discrepancies likely reflect differences in fish species, developmental stage, environmental conditions, antibiotic concentration, and exposure duration.

Dietary supplementation with *Ul*-SFE improved oxidative status and enhanced innate immune activity of carp, both in the absence and presence of antibiotics. Therefore, fish receiving *Ul*-SFE diets exhibited lower lipid peroxidation together with improved antioxidant capacity, suggesting that the UE supplementation contributed to maintaining redox homeostasis under chemotherapeutic challenge. Moreover, the comparatively higher lysozyme activity observed in the UE-fed groups may indicate a supportive effect on non-specific immune defenses. These findings agree with previous studies demonstrating that *Ulva lactuca* inclusion (5–10%) in the diet of juvenile *Oreochromis niloticus* improved antioxidant capacity [137] while *Ulva intestinalis* inclusion (0.25%, 0.5%, and 1%) in the diet of juvenile *Cyprinus carpio* increased lysozyme activity, total protein, and total immunoglobulin [119]. Such beneficial effects are largely associated with the rich biochemical profile of macroalgae, including sulfated polysaccharides, polyphenols, pigments, vitamins, and trace minerals, compounds known to neutralize reactive oxygen species and modulate immune-related pathways [138,139,140].

### 4.6. Multivariate Biochemical Profiling and Mechanistic Reconciliation of the FF/OTC Dissociation

Principal component analysis (PCA) of the extended biochemical panel (including MDA, TAC; and LYZ, in addition to the metabolic parameters discussed above) offers a multivariate visualization consistent with the univariate findings, helping to summarize the overall pattern of physiological response across treatment groups. The first principal component (PC1), accounting for 48.0% of total variance, was defined primarily by opposing loadings of MDA on one pole and TP, TAC, LYZ, and ALB on the other, broadly reflecting a composite oxidative-stress/antioxidant–immune status axis. Group centroids were ordered along this axis as C + OTC (most negative) < C + FF < U + OTC < U + FF ≈ 0 < C < U (most positive), consistent with the univariate hepatorenal and metabolic stress signature described above (elevated ALT, BUN, CRE, AMYL, CHOL and TBIL, together with depressed TP and GLOB in C + OTC), and in line with previous reports of pro-oxidant and immunosuppressive effects of OTC in rainbow trout [141,142]. The intermediate position of U + OTC relative to C + FF on PC1 is consistent with the diet-dependent buffering effect already suggested by the univariate data. The second principal component (PC2, 12.4% of variance), defined by opposing loadings of Ca, GLU and TBIL against AMYL and CHOL, separated the two antibiotics along a distinct axis: C + FF projected most strongly onto the Ca/GLU/TBIL pole, consistent with the univariate finding of elevated plasma Ca in both FF-treated groups. This pattern is plausibly related to the calcium-chelating chemistry documented for tetracycline-class antibiotics, which form stable, poorly soluble complexes with divalent cations (Ca^2+^, Mg^2+^) [143]—a property not shared by florfenicol, an amphenicol. While the separation of OTC and FF onto distinct principal components indicates that their associated biochemical response patterns differ in this dataset, this multivariate pattern should be interpreted as descriptive rather than mechanistically confirmatory; it is consistent with, but does not by itself establish, qualitatively distinct physiological mechanisms for the two antibiotics. This distinction helps explain the apparent mismatch between growth performance and biochemistry. The blood biochemistry and PCA data mainly capture hepatic, renal, and systemic oxidative/immune status—compartments where OTC produced greater variation, consistent with its cation-chelating pharmacology [143] and its previously reported pro-oxidant action in fish [141,142]. Growth performance, however, depends heavily on digestive and gut-microbial processes, which were not assessed here. Nevertheless, has been demonstrated that FF triggered intestinal dysbiosis and impaired mucosal function in cultured fish even after short-term exposure, with substantial reductions in microbial gene abundance and gut barrier integrity [144,145]. Since gut microbiota play a direct role in nutrient digestion and absorption, an FF-driven disruption of this compartment could explain slightly lower performance under florfenicol, despite its comparatively milder systemic biochemical footprint. Because no gut-histological or microbiota data were collected in the present study, this explanation should be regarded as a plausible working hypothesis rather than a confirmed mechanism, and warrants targeted verification (for instance through intestinal histomorphometry, digestive enzyme assays, or 16S rRNA-based microbiota profiling) in future work.

## 5. Conclusions

Overall, both antibiotics, administered at their respective established therapeutic doses used in standard aquaculture practice, induced metabolic, oxidative, and immune disturbances in juvenile common carp (*Cyprinus carpio*). *Ul*-SFE supplementation not only enhanced growth performance but also contributed to greater physiological stability under antibiotic challenge, by reducing oxidative stress, supporting antioxidant and immune responses, and partially attenuating liver- and kidney-related metabolic alterations. These findings support the hypothesis that Ulva-derived bioactive compounds can help alleviate antibiotic-associated stress in carp, and further highlight the potential of macroalgae extracts as functional feed ingredients for nutritional strategies aimed at minimizing the adverse metabolic, oxidative, and immunological effects of therapeutic-dose antibiotic administration in aquaculture.

## Figures and Tables

**Table 1:** HPLC profile of the *Ul*-SFE extract and health benefits of bioactive compounds.

No.	Identified Compound	Concentration, ng/g d.w. Extract	Biological Activity	References
1.	Epigallocatechin	918.63 ± 0.31	Reduces systemic LDL cholesterol oxidation. It acts as a chemopreventive and cardioprotective agent.	[52,53,54]
2.	Caffeic acid	134.43 ± 0.01	It presents powerful antioxidant, anti-inflammatory, antidiabetic, and anticancer capabilities.	[52,53,55]
3.	Protocatechuic acid	251.49 ± 0.23	Imparts profound antimicrobial and chemopreventive effects.	[52]
4.	Vanillic acid	122.19 ± 0.04	Provides hepatoprotective, cardioprotective, and neuroprotective activity. It mitigates structural tissue damage caused by systemic oxidative stress by modulating intracellular calcium levels and reducing inflammatory cytokine responses.	[52,56]
5.	Syringic acid	traces	Delivers targeted antidiabetic by improving insulin sensitivity and hepatoprotective functions by safeguarding liver hepatocytes against toxin-induced cell damage.	[57]
6.	Ferulic acid	traces	Functions as an acute anti-inflammatory and neuroprotective agent. It helps in the respiratory process by suppressing pro-inflammatory cascades.	[58,59,60]
7.	Hesperidin	traces	Present a strong vascular and capillary protection with anti-inflammatory and anti-hyperlipidemic effects.	[52,61]
8.	Epicatechin gallate	626.70 ± 0.63	Strong antibacterial by altering the structural integrity of cell walls in resistant bacteria.	[52,62]
9.	Sinapic acid	51.87 ± 0.02	Present anti-anxiety, anti-inflammatory, and antimicrobial activities. It neutralizes free radicals in highly dynamic environments and supports the overall shelf life and stability of the macroalgal mixture.	[63]
10.	Quercetin 3-diglucoside	252.98 ± 0.96	Acts as an antioxidant by its prolonged radical-scavenging effect.	[64]
11.	Quercetin	220.77 ± 0.22	It neutralizes reactive oxygen species (ROS), inhibits lipid peroxidation, and acts as an antioxidant, anti-diabetic, and anti-inflammatory.	[65,66,67]
12.	Luteolin	149.37 ± 0.08	Presents neuroprotective potential	[53]
13.	Kaempferol	436.89 ± 0.60	Anti-inflammatory properties Anti-cancer potential	[53,55,66,68]
14.	Apigenin	149.31 ± 0.20	Alongside quercetin has synergic enzyme inhibition on α-amylase and glucosidase. Plays an important role in antimicrobial activity due to the disruption of bacterial cell membranes and binds with extracellular cell wall proteins.	[69]

**Table 2:** GC-MS profiling and biological activities of bioactive compounds in *Ul*-SFE extract.

Peak	Retention Time	Identified Compound	Individual Peak Area (×10^6^)	Individual Relative Area, % ^a^	Probability, % ^b^	*m*/*z*	NIST Kovats Retention Index (RIL) ^c^	Biological Activity	References
1.	14.50	8-Hepta-decane	16.40	6.89	89.00	41, 43, 55, 57, 69, 83, 97	1700	Antimicrobial, antifungal; volatile algae aroma or defense agent	[74]
2.	18.52	Manool	1.53	0.64	69.90	137,81, 95, 41, 69, 257	2040–2060	Cytotoxic (anti-cancer potential), anti-inflammatory	[75]
3.	18.98	Phytol	50.57	21.23	91.50	41, 43, 55, 71, 81, 95, 123	2110–2140	Antioxidant, antimicrobial, anti-inflammatory, chlorophyll building block	[71,72,73]
4.	19.66	Sesqui-terpene esters	1.90	0.80	73.10	69, 41, 93, 79, 105, 119	1600–1900	Antimicrobial barrier, signaling molecule of the macroalga	[53]
5.	22.46	2-Oleyloxy-1-ethanol	3.48	1.46	71.20	41, 55, 67, 69, 82, 96, 250	2200–2400	Cell membrane protection (antiaging), Immunomodulatory effect	[76,77,78]
6.	22.57	Methyl palmitate	3.76	1.58	69.10	29, 43, 57, 74, 84, 98, 239	1875–1885	Antioxidant, antibacterial	
7.	24.37	Squalene	1.69	0.71	77.40	69,81, 136, 341, 410	2800–2850	Antioxidant, cytotoxic (anti-tumor), cell-protective	[79,80,81]
8.	26.53	Phytosterol isomer	1.41	0.59	60.00	43, 55, 105, 386, 95, 81, 145	3100–3300	Structural component of the algal cell membrane	[77,78]

^a^ Values were calculated to the total integrated peak area. ^b^ Scores above 80% indicate reliable structural assignments. ^c^ RI_L_—Kovats Retention Index according to official NIST Chemistry WebBook (NIST Chemistry WebBook, NIST Standard Reference Database Number 69, https://webbook.nist.gov/chemistry/, accessed on 16 June 2026), and from the literature [82].

**Table 3:** Growth performance indicators of carp fed Control and Ul-SFE supplemented diets across experimental periods (mean ± SD).

Experimental Periods	Experimental Variants	FCR	SGR (% Day^−1^)	PER
P1	C (Control diet)	1.866 ± 0.282	1.003 ± 0.147	1.701 ± 0.262
	U (Ul-SFE diet)	1.689 ± 0.019	1.074 ± 0.012	1.851 ± 0.021
P2	C (Control diet)	1.653 ± 0.120	0.966 ± 0.073	1.898 ± 0.139
	U (Ul-SFE diet)	1.526 ± 0.069	1.017 ± 0.043	2.050 ± 0.091
P3	C (Control diet)	1.155 ± 0.036	1.579 ± 0.068	2.707 ± 0.083
	U (Ul-SFE diet)	1.086 ± 0.008	1.630 ± 0.026	2.877 ± 0.020
P4	C (Control diet)	1.458 ± 0.041	1.192 ± 0.038	2.144 ± 0.059
	U (Ul-SFE diet)	1.327 ± 0.114	1.266 ± 0.096	2.366 ± 0.197
P1-P4	C (Control diet)	1.484 ± 0.018	1.154 ± 0.019	2.106 ± 0.011
	U (Ul-SFE diet)	1.375 ± 0.023	1.207 ± 0.030	2.275 ± 0.088
Diet		F(1,4) = 11.58, *p* = 0.027	F(1,4) = 16.51, *p* = 0.015	F(1,4) = 20.41, *p* = 0.011
Period		F(3,12) = 27.88, *p* < 0.001	F(3,12) = 74.39, *p* < 0.001	F(3,12) = 63.40, *p* < 0.001
Diet × Period		F(3,12) = 0.26, *p* = 0.851	F(3,12) = 0.79, *p* = 0.521	F(3,12) = 0.11, *p* = 0.953

Values are presented as mean ± SD (n = 3 replicate tanks/diet/period). Diet, Period, and Diet × Period effects were tested using a mixed-design (split-plot) ANOVA, with diet as the between-subject factor and period as the within-subject repeated factor.

**Table 4:** Growth performance of carp during the 10-day antibiotic phase (mean ± SD).

Parameter	C	C + FF	C + OTC	U	U + FF	U + OTC	D	A	D × A
TWG (g)	258.50 ± 9.41 ^a^	231.20 ± 10.21 ^b^	238.33 ± 12.11 ^b^	262.66 ± 4.23 ^a^	248.21 ± 13.11 ^b^	255.24 ± 25.12 ^a^	*	*	ns
TFI (g)	408.91 ± 2.12 ^a^	385.10 ± 5.76 ^b^	387.23 ± 7.61 ^b^	397.42 ± 7.31 ^ab^	405.54 ± 5.09 ^a^	401.12 ± 11.16 ^ab^	*	ns	**
FCR	1.59 ± 0.07 ^b^	1.67 ± 0.05 ^a^	1.63 ± 0.05 a ^b^	1.51 ± 0.06 c	1.62 ± 0.09 ^b^	1.56 ± 0.12 ^b^	*	***	ns
SGR (%/day)	1.73 ± 0.04 ^b^	1.65 ± 0.08 ^d^	1.69 ± 0.09 ^c^	1.80 ± 0.06 ^a^	1.68 ± 0.08 ^cd^	1.74 ± 0.10 ^b^	**	***	ns
PER	1.97 ± 0.04	1.87 ± 0.06	1.92 ± 0.06	2.09 ± 0.07 ^a^	1.91 ± 0.13	1.99 ± 0.25	**	**	ns
Survival (%)	100	90	97	100	97	100			

Values are presented as mean ± SD (n = 3 replicate tanks/group). Within a row, means with different superscript letters differ significantly (Tukey HSD, *p* < 0.05). Diet (D), Antibiotic (A), and D × A columns show the significance of the two-way ANOVA main effects and interaction: ns = not significant (*p* ≥ 0.05), * *p* < 0.05, ** *p* < 0.01, *** *p* < 0.001.

**Table 5:** Plasma biochemical parameters in experimental groups (mean ± SD).

Parameter	C	C + FF	C + OTC	U	U + FF	U + OTC
TP (g dL^−1^)	3.70 ± 0.26 ^bc^	3.63 ± 0.16 ^c^	3.39 ± 0.20 ^d^	3.91 ± 0.23 ^a^	3.89 ± 0.06 ^ab^	3.65 ± 0.18 ^c^
ALB (g dL^−1^)	1.93 ± 0.09 ^b^	1.86 ± 0.08 ^c^	1.86 ± 0.04 ^c^	2.02 ± 0.07 ^a^	1.94 ± 0.04 ^b^	1.90 ± 0.03 ^bc^
GLOB (g dL^−1^)	1.77 ± 0.23 ^b^	1.76 ± 0.12 ^ab^	1.54 ± 0.17 ^c^	1.89 ± 0.16 ^ab^	1.95 ± 0.07 ^a^	1.83 ± 0.17 ^ab^
A/G ratio	1.11 ± 0.17 ^ab^	0.95 ± 0.06 ^a^	1.22 ± 0.13 ^b^	1.07 ± 0.07 ^ab^	1.00 ± 0.05 ^a^	1.05 ± 0.10 ^ab^
TBIL (mg dL^−1^)	0.21 ± 0.04 ^bc^	0.25 ± 0.04 ^ab^	0.28 ± 0.03 ^a^	0.20 ± 0.03 ^c^	0.24 ± 0.04 ^ab^	0.21 ± 0.08 ^bc^
ALT (U L^−1^)	52.60 ± 3.72 ^c^	59.60 ± 6.33 ^b^	66.60 ± 2.59 ^a^	56.20 ± 2.18 ^bc^	57.80 ± 1.49 ^b^	59.40 ± 4.12 ^b^
ALP (U L^−1^)	35.40 ± 2.35 ^a^	36.60 ± 2.23 ^a^	37.61 ± 1.99 ^a^	35.87 ± 1.81 ^a^	37.27 ± 3.10 ^a^	36.47 ± 1.81 ^a^
BUN (mg dL^−1^)	6.22 ± 0.11 ^c^	7.19 ± 1.28 ^ab^	7.53 ± 0.58 ^a^	6.10 ± 0.14 ^c^	6.27 ± 0.54 ^c^	6.68 ± 0.92 ^bc^
CREA (mg dL^−1^)	0.61 ± 0.13 ^ab^	0.69 ± 0.13 ^a^	0.71 ± 0.20 ^a^	0.51 ± 0.05 ^b^	0.64 ± 0.06 ^a^	0.59 ± 0.06 ^ab^
AMYL (U L^−1^)	168.40 ± 27.93 ^d^	219.00 ± 15.83 ^c^	256.00 ± 16.70 ^a^	221.05 ± 16.21 ^bc^	212.80 ± 31.88 ^c^	246.40 ± 31.54 ^ab^
GLU (mg dL^−1^)	83.99 ± 9.23 ^b^	96.24 ± 4.31 ^a^	101.42 ± 4.53 ^a^	83.26 ± 8.37 ^b^	88.18 ± 3.38 ^b^	82.32 ± 4.37 ^b^
CHOL (mg dL^−1^)	255.53 ± 22.59 ^de^	274.09 ± 15.87 ^bc^	295.25 ± 9.17 ^a^	241.81 ± 13.92 ^e^	265.59 ± 13.36 ^cd^	287.11 ± 8.58 ^b^
Ca (mg dL^−1^)	7.76 ± 0.71 ^bc^	8.92 ± 0.74 ^a^	7.75 ± 1.14 ^bc^	7.05 ± 0.88 ^cd^	8.63 ± 1.20 ^ab^	6.53 ± 0.17 ^d^
P (mg dL^−1^)	7.50 ± 0.45 ^b^	7.65 ± 1.30 ^b^	8.20 ± 0.75 ^b^	10.18 ± 1.78 ^a^	7.99 ± 0.86 ^b^	9.91 ± 1.10 ^a^

Values are presented as mean ± SD (n = 5 fish/group). Different superscript letters within the same column indicate significant differences between groups (Tukey HSD, *p* < 0.05).

**Table 6:** Results of the two-way ANOVA evaluating the effects of diet, therapeutic-dose antibiotic administration, and their interaction on plasma biochemical parameters of common carp.

Parameter	Diet (*p*)	η^2^*p*	Antibiotic (*p*)	η^2^*p*	Diet × Antibiotic (*p*)	η^2^*p*
TP (g dL^−1^)	*p* < 0.001	0.30	*p* < 0.001	0.31	*p* = 0.838	0.00
ALB (g dL^−1^)	*p* < 0.001	0.27	*p* < 0.001	0.33	*p* = 0.209	0.04
GLOB (g dL^−1^)	*p* < 0.001	0.23	*p* < 0.001	0.26	*p* = 0.033	0.08
A/G ratio	*p* = 0.017	0.07	*p* < 0.001	0.31	*p* < 0.001	0.17
TBIL (mg dL^−1^)	*p* = 0.002	0.11	*p* < 0.001	0.17	*p* = 0.010	0.10
ALT (U L^−1^)	*p* = 0.026	0.06	*p* < 0.001	0.48	*p* < 0.001	0.27
ALP (U L^−1^)	*p* = 0.996	0.00	*p* = 0.032	0.08	*p* = 0.243	0.03
BUN (mg dL^−1^)	*p* < 0.001	0.17	*p* < 0.001	0.24	*p* = 0.067	0.06
CREA (mg dL^−1^)	*p* < 0.001	0.13	*p* = 0.002	0.14	*p* = 0.519	0.02
AMYL (U L^−1^)	*p* = 0.019	0.06	*p* < 0.001	0.49	*p* < 0.001	0.27
GLU (mg dL^−1^)	*p* < 0.001	0.38	*p* < 0.001	0.31	*p* < 0.001	0.29
CHOL (mg dL^−1^)	*p* = 0.002	0.11	*p* < 0.001	0.60	*p* = 0.713	0.01
Ca (mg dL^−1^)	*p* < 0.001	0.16	*p* < 0.001	0.42	*p* = 0.126	0.05
P (mg dL^−1^)	*p* < 0.001	0.35	*p* < 0.001	0.20	*p* < 0.001	0.16

*p*-values, and partial eta squared (η^2^*p*) are presented for each main effect and interaction. Based on conventional benchmarks, partial η^2^ values of approximately 0.01, 0.06 and 0.14 indicate small, moderate and large effect sizes, respectively.

**Table 7:** Oxidative stress and innate immune parameters in experimental groups (mean ± SD).

Group	LYZ (U/mg Solid)	MDA (nmoles/mL Plasma)	TAC (mM TROLOX)
C	25.61 ± 0.66 ^b^	7.60 ± 0.35 ^c^	25.61 ± 0.56 ^b^
C + FF	23.00 ± 0.44 ^c^	9.90 ± 1.30 ^b^	22.81 ± 0.46 ^d^
C + OTC	22.70 ± 0.56 ^c^	11.22 ± 1.08 ^a^	21.46 ± 0.34 ^e^
U	30.81 ± 2.62 ^a^	5.29 ± 0.41 ^d^	27.16 ± 0.89 ^a^
U + FF	24.62 ± 0.50 ^b^	7.57 ± 0.60 ^c^	24.43 ± 0.61 ^c^
U + OTC	22.91 ± 0.32 ^c^	8.33 ± 0.73 ^c^	22.48 ± 0.68 ^d^
Two-way ANOVA summary			
Parameter	Diet (Ulva)	Antibiotic	Diet × Antibiotic
MDA	*p* < 0.01	*p* < 0.01	*p* = 0.309
TAC	*p* < 0.01	*p* < 0.01	*p* = 0.129
LYZ	*p* < 0.01	*p* < 0.01	*p* < 0.001

Different superscript letters within the same column indicate significant differences between groups according to Tukey’s HSD test (*p* < 0.05).

## Data Availability

The original contributions presented in this study are included in the article/Supplementary Material. Further inquiries can be directed to the corresponding author.

## Supplementary Materials

The following supporting information can be downloaded at: https://www.mdpi.com/article/10.3390/antiox15091171/s1.
