# Supplementary material for: The Inclusion of Supercritical Fluid Extract of Ulva lactuca in the Diet of Common Carp (Cyprinus carpio) Enhances Physiological Resilience and Antioxidant Status During Therapeutic-Dose Antibiotic Administration
Source: Antioxidants (Basel). 2026 Sep 15;15(9):1171. doi: 10.3390/antiox15091171 (PMC13603286; doi:10.3390/antiox15091171)
Supplement: Supplementary file 1 [file antioxidants-15-01171-s001.zip › antioxidants-4504487-supplementary.pdf]

antioxidants

MDPI

Supplementary Information

The Inclusion of Supercritical Fluid Extract of *Ulva lactuca* in the Diet of Common Carp (*Cyprinus carpio*) Enhances Physiological Resilience and Antioxidant Status During Therapeutic-Dose Antibiotic Administration

Alina Nicoleta Macoveiu (Dobre) [1,2], Liliana Mihalcea [1,*], Dana Moraru [1], Nicoleta Bălan [1], Mirela Crețu [3], Angelica Docan [1], Iulia Grecu [1], Cristian Rîmniceanu [1], Daniela Ionela Istrati [1], Floricel Maricel Dima [2,4] and Lorena Dediu [1,*]

[1] Faculty of Food Science and Engineering, Dunarea de Jos University of Galati, 111 Domneasca Street, 800201 Galați, Romania; adobre14@gmail.com (A.N.M.); dana.moraru@ugal.ro (D.M.); nicoleta.balan@ugal.ro (N.B.); angelica.docan@ugal.ro (A.D.); iulia.grecu@ugal.ro (I.G.); cristian.rimniceanu@ugal.ro (C.R.); daniela.istrati@ugal.ro (D.I.I.)

[2] Institute of Research and Development for Aquatic Ecology, Fishing and Aquaculture, 54 Portului Street, 800211 Galați, Romania; dima.floricel.maricel@asas-icdeapa.ro

[3] Transborder Faculty, Dunărea de Jos University of Galați, 47 Domnească Street, 800008 Galați, Romania; mirela.cretu@ugal.ro

[4] Faculty of Engineering and Agronomy in Braila, Dunarea de Jos University of Galati, 29 Calea Calarașilor Street, 810017 Brăila, Romania

* Correspondence: liliana.gitin@ugal.ro (L.M.); lorena.dediu@ugal.ro (L.D.)

Table S1. *Proximate composition and micronutrient content of the basal commercial diet (Coppens Premium Select 2.0, Coppens International, Nijkerk, The Netherlands), as declared by the manufacturer.*

Component	Content
Crude protein	32%
Crude fat	15%
Crude fiber	1.90%
Ash	9.40%
Phosphorus	1.24%
Calcium	1.10%
Sodium	0.20%
Vitamin A	10,000 IU/kg
Vitamin D3	2,103 IU/kg
Iron	40 mg/kg
Iodine	23 mg/kg

 https://doi.org/ 10.3390/antiox15091171

Copper	33 mg/kg
Manganese	13.3 mg/kg
Zinc	40 mg/kg
Selenium	0.1 mg/kg

Table S2. Experimental groups used to evaluate the effects of dietary *Ulva lactuca* extract supplementation and therapeutic-dose antibiotic administration in common carp.

Experimental group	Phase 1: 10 weeks	Phase 2: 10 days	Antibiotic dose
C	Conventional commercial diet	No antibiotic	-
C+FF	Conventional commercial diet	Florfenicol	15 mg kg^{-1} body weight day^{-1}
C+OTC	Conventional commercial diet	Oxytetracycline	75 mg kg^{-1} body weight day^{-1}
U	Diet supplemented with *Ul*-SFE, 50 mg kg^{-1} feed	No antibiotic	-
U+FF	Diet supplemented with *Ul*-SFE, 50 mg kg^{-1} feed	Florfenicol	15 mg kg^{-1} body weight day^{-1}
U+OTC	Diet supplemented with *Ul*-SFE, 50 mg kg^{-1} feed	Oxytetracycline	75 mg kg^{-1} body weight day^{-1}

HPLC Analysis of the *Ul*-SFE extract

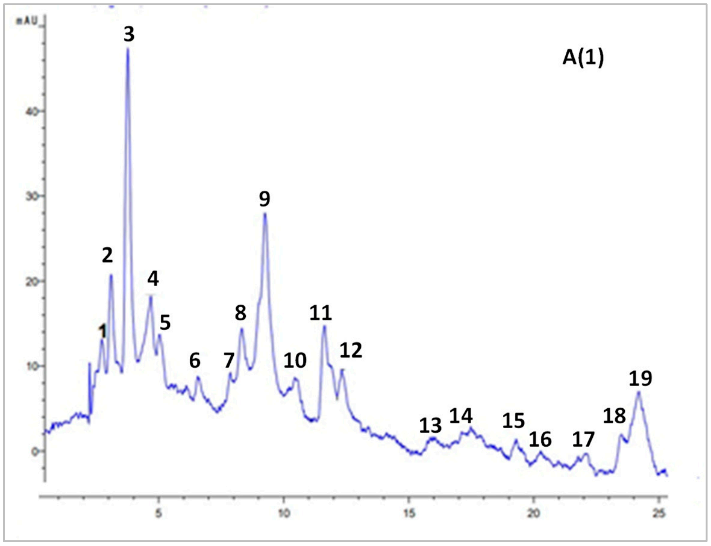

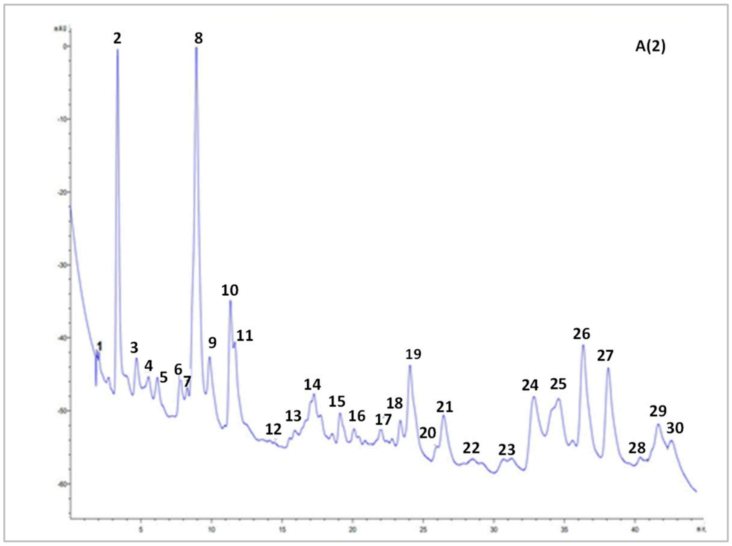

Figure S1. HPLC chromatograms for the hydrophilic profile of the Ul–SFE extract

A(1) 280 nm: 1 – protocatechuic acid, 2-3 – unidentified compounds, 4 – vanillic acid, 5 – unidentified compound, 6 – epicatechin gallate, 7 - 9 – unidentified compounds; 10 – sinapic acid, 5 – unidentified compound, 12 – quercetin 3-glucoside, 13 - 14 – unidentified compounds; 15 – quercetin, 16 - 18 – unidentified compounds; 19 – kaempferol. A(2) 320 nm: 1 – epigallocatechin, 2 - 3 – unidentified compounds, 4 – syringic acid, 5 – epicatechin gallate, 6 – unidentified compounds; 7 – ferulic acid, 8 - 11 – unidentified compounds, 12 – hesperidin, 13 - 14 – unidentified compound, 15 – quercetin, 16 – caffeic acid; 17 – luteolin, 18 - 19 – unidentified compounds; 20 – apigenin, 21 - 30 – unidentified compounds.

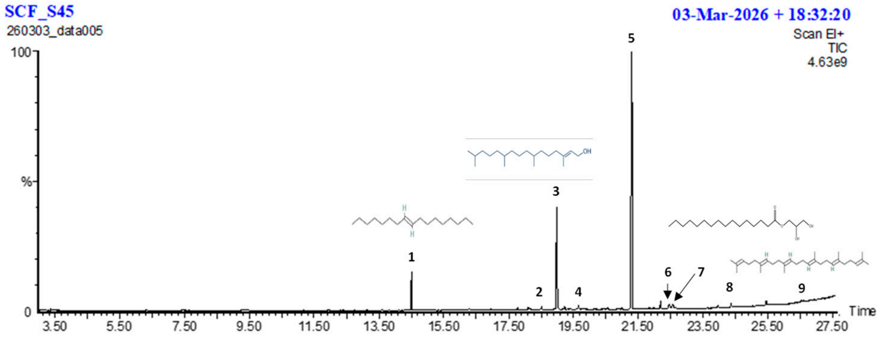

Figure S2. GC-MS chromatogram for the S45 fraction of *Ul*-SFE extract (1 – 8-Heptadecene; 2 – Labdane diterpene; 3 – Phytol; 4 – Sesquiterpene esters; 5 – not identified; 6 – 2-oleyloxy-1-ethanol; 7 – 2-monopalmitin; 8 – squalene; 9 – phytosterol isomer)

https://doi.org/ 10.3390/antiox15091171
